# Preoperative Assessment of Neural Elements in Lumbar Spinal Stenosis by Upright Magnetic Resonance Imaging: An Implication for Routine Practice?

**DOI:** 10.7759/cureus.2440

**Published:** 2018-04-06

**Authors:** Gernot Lang, Marco Vicari, Alexander Siller, Eva J Kubosch, Juergen Hennig, Norbert P Südkamp, Kaywan Izadpanah, David Kubosch

**Affiliations:** 1 Department of Orthopedic and Trauma Surgery, Medical Center - Albert-Ludwigs-University of Freiburg, Faculty of Medicine, Albert-Ludwigs-University of Freiburg, Germany; 2 Fraunhofer Institute for Medical Image Computing Mevis, Fraunhofer Institute for Medical Image Computing Mevis, Bremen, Germany; 3 Medical Physics, Department of Radiology, Medical Center - Albert-Ludwigs-University of Freiburg, Faculty of Medicine, Albert-Ludwigs-University of Freiburg, Germany

**Keywords:** upright mri, neural stenosis, degeneration, lumbar, decompression, upright mri, spine, mri, surgery

## Abstract

Introduction

Lumbar spinal stenosis (LSS) is a kinetic-dependent disease typically aggravating during spinal loading. To date, assessment of LSS is usually performed with magnetic resonance imaging (MRI). However, conventional supine MRI is associated with significant drawbacks as it does not truly reflect physiological loads, experienced by discoligamentous structures during erect posture. Consequently, supine MRI often fails to reveal the source of pain and/or disability caused by LSS.

The present study sought to assess neural dimensions via MRI in supine, upright, and upright-hyperlordotic position in order to evaluate the impact of patient positioning on neural narrowing. Therefore, radiological measures such as neuroforaminal dimensions, central canal volume, sagittal listhesis, and lumbar lordosis at spinal level L4/5 were extracted and stratified according to patient posture.

Materials and methods

Overall, 10 subjects were enclosed in this experimental study. MRI was performed in three different positions: (1) 0° supine (SP), (2) 80° upright (UP), and (3) 80° upright + hyperlordotic (HY) posture. Upright MRI was conducted utilizing a 0.25T open-configuration scanner equipped with a rotatable examination bed allowing for true standing MRI. Radiographic outcome of upright MRI imaging was extracted and evaluated according to patient positioning.

Results

Upright MRI-based assessment of neural dimensions was successfully accomplished in all subjects. Overall, radiographic parameters revealed a significant decrease of neural dimensions from supine to upright position: Specifically, mean foraminal area decreased from SP to UP by 13.3% (P ≤ 0.05) as well as from SP to HY position by 21% (P ≤ 0.05). Supplementation of hyperlordosis did not result in additional narrowing of neural elements (P ≥ 0.05). Furthermore, central canal volume revealed a decrease of 7% at HY and 8% at UP compared to SP position (P ≥ 0.05). Assessment of lumbar lordosis yielded in a significant increase when assessed at HY (+22.1%) or UP (+8.7%) compared to SP (P ≤ 0.05).

Conclusions

Our data suggest that neuroforaminal dimensions assessed by conventional supine MRI are potentially overestimated in patients with LSS. Especially, in patients having occult disease not visualized on conventional imaging modalities, upright MRI allows for a precise, clinically relevant, and at the same time non-invasive evaluation of neural elements in LSS when neural decompression is considered.

## Introduction

Lumbar spinal stenosis (LSS) as well as degenerative spondylolisthesis (DS) is frequent and disabling condition mostly occurring in the elderly [1]. LSS is associated with central canal-, foraminal-, and/or lateral recessus narrowing due to age-related degenerative alterations of the spinal alignment, including intervertebral disc bulging, spondylarthrosis, and hypertrophic ligamenta flava and facet joints. Ultimately, LSS potentially results in progressive circumferential central canal and neuroforaminal compression, indicated by severe radicular and lower back pain, muscular fatigue, and finally intermittent spinal claudication. Posture influences neural dimensions and symptoms may only be present or aggravate in the upright position.

Most commonly, LSS affects the spinal segments L4/5 [1]. If conservative therapy fails, surgical treatment by means of direct or indirect decompression has to be considered [2, 3]. For successful neural decompression, precise diagnostics, elimination of differential diagnoses, and the choice of a sufficient surgical strategy are detrimental. Therefore, patients should undergo a standardized diagnostic algorithm including thorough clinical examination, conventional standing X-rays as well as magnetic resonance imaging (MRI), which is routinely performed by utilization of a pillow under the patient’s knees leading to relaxation of the psoas muscle and flexion of the lumbar spine. However, supine MRI often correlates insufficiently with the patient’s symptomatology, most probably because LSS correlates with positional dependence and dynamic changes of neural elements and their surrounding tissues. Subsequently, dynamic pathologies such as LSS may remain undiagnosed in the absence of axial loading as lumbar extension results in a maximization of neural dimensions, potentially hiding foraminal and/or central canal narrowing due to the unphysiologic method of supine image acquisition [1, 4, 5].

Dynamic upright MRI technology has therefore gained scientific interest, aiming to overcome current diagnostic barriers in order to provide a reliable tool for the assessment of neural dimensions under physiologic weight-bearing conditions [6]. Splendiani et al. published (to our best knowledge) the largest study on upright MRI, comparing morphologic differences between supine and upright MRI in 4305 patients having low back pain. In 67% of patients, upright MRI revealed significant alterations compared to supine imaging [7, 8]. Previously unknown disc protrusion, spinal canal stenosis, translational vertebral movement, and lumbar lordosis were found to be more significant in upright than in supine MRI. Especially in complex cases with position-dependent impairment, upright MRI may facilitate preoperative evaluation of neural elements in order to assist in surgical decision-making, preoperative planning, and potentially optimize clinical outcomes. Nevertheless, previous investigations of spinal canal imaging under axial load were mostly cadaver studies [9]. Additionally, recent studies on axially loaded supine MRI failed to reflect postural spinal alterations [10]. Only few studies analyzed segmental listhesis or dynamic changes of central canal dimensions [11].

Therefore, the present study sought to evaluate neural elements and spinal alignment parameters in (1) conventional supine, and upright position, (2) alone, and (3) combined with hyperextension at spinal level L4/5 by utilization of a positional MRI scanner in patients with LSS.

We hypothesized, (a) that conventional supine MRI at spinal level L4/5 may underestimate neural narrowing due to LSS. Secondly, we hypothesized (b) that upright MRI may provide a more accurate assessment of neural dimensions and spinal alignment compared to supine MRI.

## Materials and methods

Study population

Between 2010 and 2012, a prospective single-center study of patients undergoing transforaminal lumbar interbody fusion (TLIF) at spinal level L4/L5 as a treatment for symptomatic single or multilevel lumbar degenerative disorders was conducted. Patients had been enrolled for TLIF for various clinical indications including central canal stenosis, low-grade spondylolisthesis (Meyerding Grades I and II), facet arthropathy, foraminal stenosis, etc. (Table 1). Patients with congenital scoliosis, thoracic spinal disorders, or severe osteoporosis (Z-score -2.5 or less) were not eligible for direct decompression techniques and were excluded from this study. Furthermore, patients with a history of spinal tumor or trauma were excluded as well. Patients usually described a chronicity of symptoms ≥ 6 months containing failed conservative therapy. All patients were scheduled for TLIF L4/L5 after completing a preoperative diagnostic algorithm including conventional X-rays, periradicular infiltration of spinal level L4/5, and pain assessment while wearing a stabilizing thoracolumbar corset. Exclusion criteria were primary spondylolysis (pars interarticularis defect), previous surgical interventions, and any malignancies. Patients referred to conventional supine MRI at the Department of Trauma and Orthopedic Surgery, University Medical Center Freiburg, Germany, were asked to participate in the present experimental study, and give written consent.

**Table 1:** Baseline characteristics of patients undergoing upright MRI. N: Absolute number of patients; %: Relative number of patients in percent. ^1^Mean ± SE; ^2^Multiple answers allowed; ^3^Meyerding Grades I to II.

Parameter		N	%
Mean age at examination in years	Max: 86 Min: 61	76.7 ± 8	
Gender ^1^	Male	3	30
	Female	7	70
Pathology at spinal level L4/5^2^	Degenerative scoliosis	7	70
	Central canal stenosis	7	70
	Foraminal stenosis	3	30
	Lateral recess stenosis	1	10
	Degenerative spondylolisthesis^3^	9	90
Low back pain		10	100
Neurological deficits		5	50

Image acquisition

**Figure 1:**
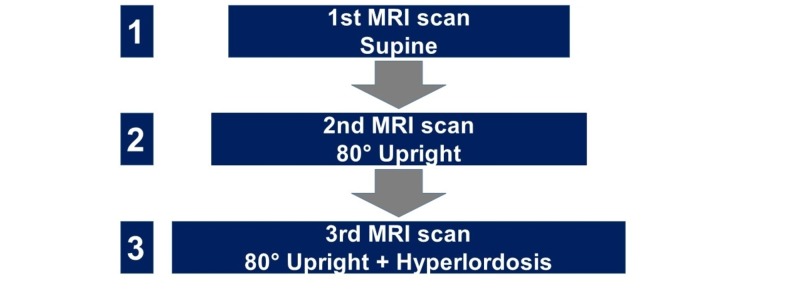
Flow chart illustrating the sequence of MRI scans in different positions. MRI: Magnetic resonance imaging.

All participants were scanned in a low-field 0.25T open-configuration scanner (G-Scan, Esaote, Genoa, Italy) equipped with a rotatable examination bed allowing for true standing MRI as described previously [6]. Assessment of neural dimensions and spinal alignment parameters were repeated blinded for previous outcome within one day. Dynamic MRI was performed in three different positions (Figure 1): (1) 0° supine, (2) 80° upright, and (3) 80° upright + hyperlordosis).

Gradient supports ±20 mT/m with a slew rate of 25 mT/m/ms. Phased array dedicated receiving coils were used. Sagittal MRI examinations included a 2D FSE T2 sequence (TR = 3350 ms, TE = 120 ms, FOV = 310 × 310 mm^2^, M = 224 × 208, TH = 4 mm, TA = 5’28”) and a 3D HYCE (balanced steady state sequence, TR = 10 ms, TE = 5 ms, FOV = 290 × 290 × 100 mm^3^, M = 232 × 206 × 28, TA = 5’29”).

Radiological evaluation

MRI sequences were evaluated as described previously [6]. Neural dimensions, such as foraminal and central canal area, were evaluated at spinal level L4/5. Medical Image Viewer Impax (Agfa HealthCare, Trier, Germany) was used for standardized assessment of neural structures. Great care was taken to avoid measurement errors due to partial volume effects caused by differences in the patients' positioning. In the sagittal view, the slice was oriented along the ground plate of L4. In the axial view, the slice was oriented alongside the middle of the vertebral body and spinal process. Comparative measurements of the spinal canal volume, spinal canal, and neuroforaminal diameter, and its area were extracted at each position. Moreover, intervertebral listhesis and segmental lordosis were recorded as shown previously [6].

Statistical analysis

Continuous variables are shown as mean ± standard error of the mean. A one-way analysis of variance (ANOVA) for repeated measurements was used to assess statistical differences. Post-hoc Bonferroni Test was used to identify differences between conditions. Analysis was considered statistically significant with a p-value ≤ 0.05. For categorical variables percentages were calculated. All analyses were performed using SPSS v22 (IBM, Armonk, NY, USA).

Ethical considerations

The study was approved by our local institutional review board and informed consent was obtained from all patients before enrolment (protocol number: 297/10).

## Results

Overall, 10 subjects were analyzed. Supine- and upright MRI-based assessment of radiographic outcome measures was successfully accomplished in all patients.

Assessment of neural dimensions

Neural dimensions by means of foraminal diameter, foraminal area, and central canal volume revealed a significant decrease from (1) supine (SP) to (2) 80° upright (UP) position or 80° upright position combined with hyperextension (HY; Table 2).

**Table 2:** Radiographic outcome of patients undergoing upright magnetic resonance imaging (MRI) at spinal level L4/5. 1: Supine MRI; 2: 80° Upright MRI; 3: 80° Upright MRI + Hyperextension; ^1^Mean ± SD; Δ1: Absolute difference between supine and 80° upright MRI; P1: Statistical significance between supine  and 80° upright MRI; Δ2: Absolute difference between supine and 80° upright MRI + Hyperextension; P2: Statistical significance between supine and 80° upright MRI + Hyperextension; Δ3: Absolute difference between 80° upright MRI and 80° upright MRI + Hyperextension; P3: Statistical significance between 80° upright MRI and 80° upright MRI + Hyperextension; CSA: Central canal area; P: P-value. P values ≤ 0.05 are considered statistically significant.

Evaluation of neural elements									
	1	2			3				
Radiographic parameter	Supine	80° upright	Δ1	P1	80° upright + hyperlordosis	Δ2	P2	Δ3	P3
Mean CSA (mm^2^)^1^	9672.9 ± 3146.9	8867.0 ± 2129.1	-805.9 (-8.3%)	1.0	8993.7 ± 1778.1	-679.2 (-7.0%)	0.862	-126.7 (-1.4%)	0.739
Mean sagittal translation L4/5^1^	3.7 ± 3.2	4.5 ± 2.8	+0.7 (+19.5%)	0.352	3.9 ± 2.1	+0.2 (+5.5%)	1.0	-0.52 (-11.7%)	0.227
Mean segmental listhesis (mm)^1^	7.1 ± 3.1	7.7 ± 2.8	+0.6 (+9.1%)	0.540	8.2 ± 2.3	+1.1 (16%)	0.123	+0.49 (+6.4%)	0.362
Mean foraminal diameter (mm)^1^	7.2 ± 1.6	6.5 ± 1.7	-0.7 (-9.9%)	0.012	6.5 ± 1.9	-0.72 (-9.9%)	0.008	0 (0%)	1.0
Mean foraminal area (mm²)^1^	97.6 ± 27.2	84.6 ± 20.8	-13.0 (-13.3%)	0.031	77.1 ± 20.3	-20.5 (-21.0%)	0.003	-7.6 (-8.9%)	0.087
Mean lumbar lordosis (°)^1^	49.2 ± 10.3	53.5 ± 13.5	+4.3 (+8.7%)	0.231	60.1 ± 12.1	+10.90 (+22.1%)	0.001	+6.6 (+12.4%)	0.002

**Figure 2:**
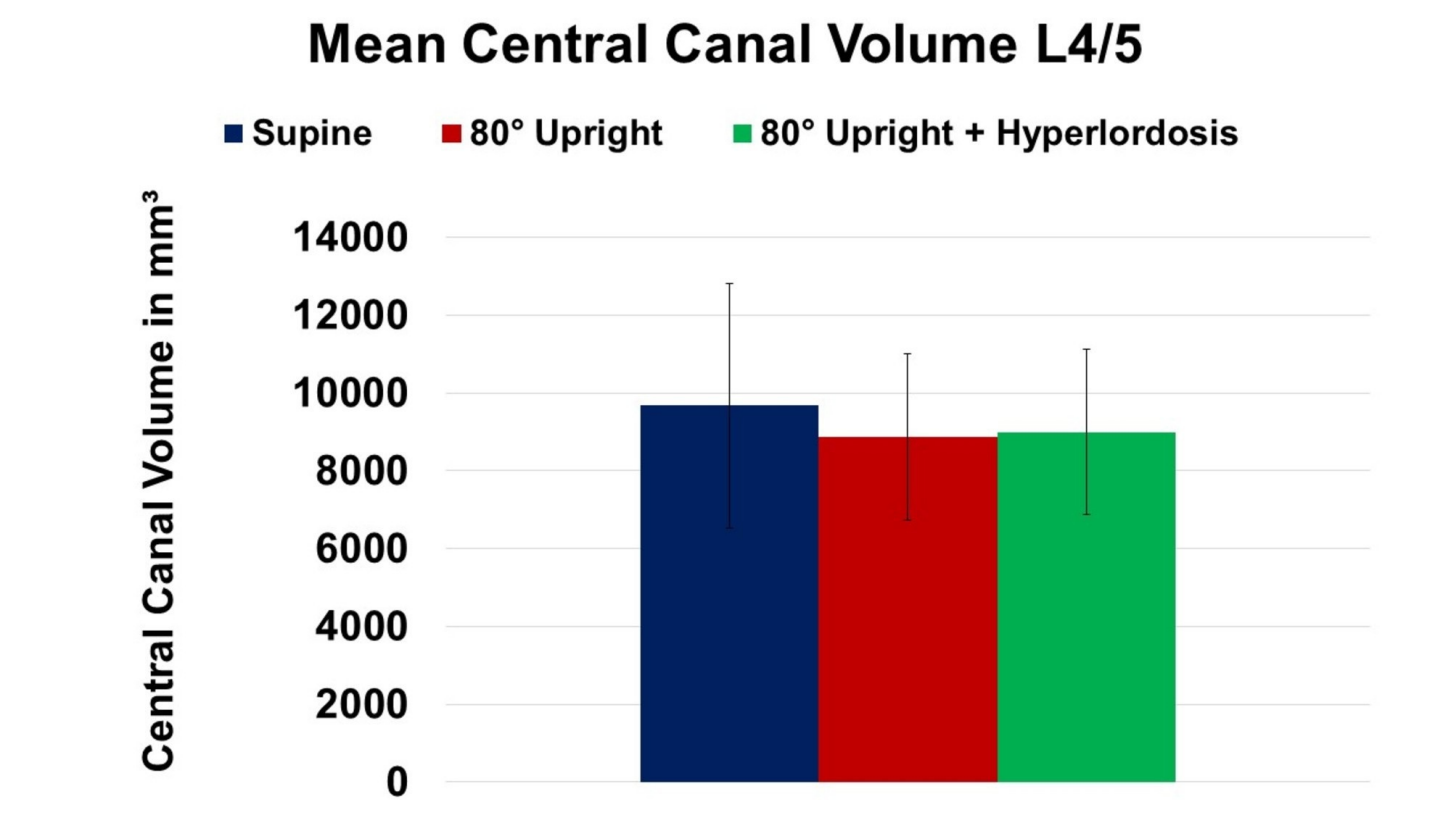
Change of mean central canal volume at spinal level L4/5 in supine, 80° upright, and 80° upright combined with hyperlordosis position.

Specifically, central canal volume decreased from supine to 80° upright by -8% and from supine to 80° upright combined with hyperlordotic posture by -7% (Figure 2).

**Figure 3:**
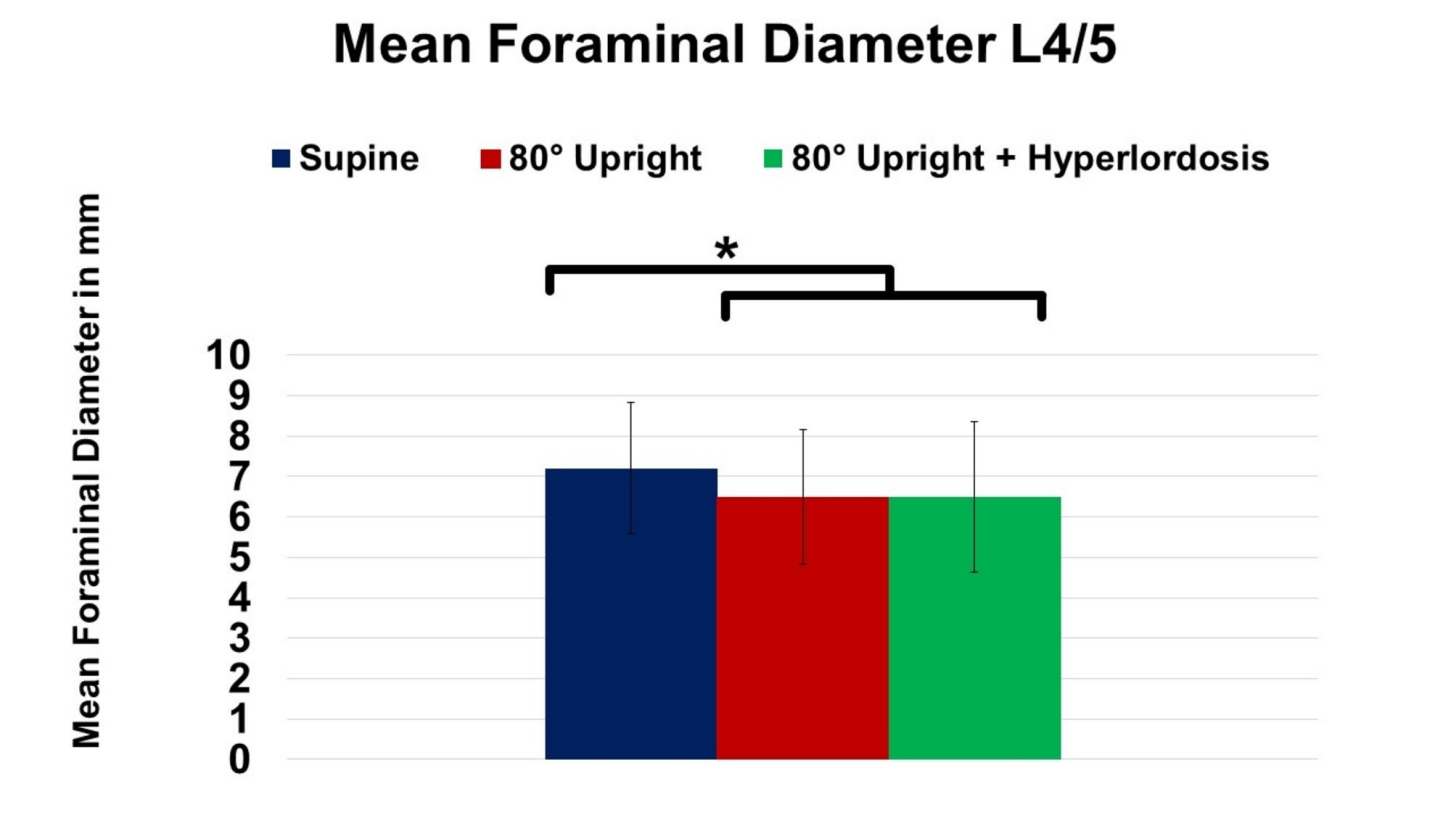
Change of mean foraminal diameter at spinal level L4/5 in supine, 80° upright, and 80° upright combined with hyperlordosis position. *P ≤ 0.05

Significant differences in the assessment of central canal volume between UP and HY were not detected (P ≥ 0.05). Furthermore, mean foraminal diameter at L4/5 decreased from SP to UP by 10% (P = 0.012; Figure 3). Addition of hyperlordosis to erect imaging did not further decrease foraminal diameters compared to UP (P ≥ 0.05).

**Figure 4:**
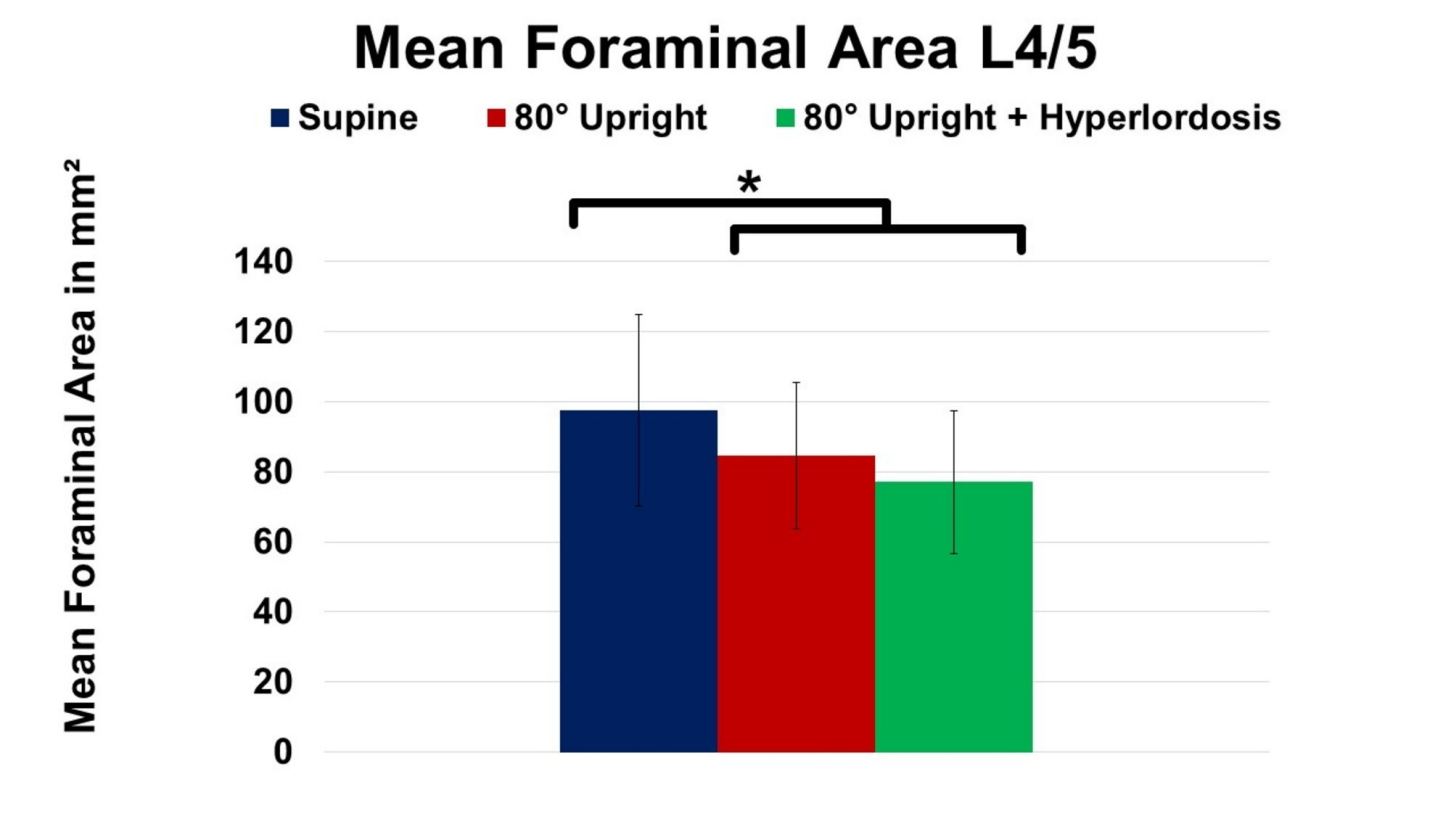
Change of mean foraminal area at spinal level L4/5 in supine, 80° upright, and 80° upright combined with hyperlordosis position. *P ≤ 0.05

Subsequently, mean foraminal area at L4/5 also revealed significant decline at UP imaging when compared to supine (SP) imaging (-13%; P = 0.031). Supplementation of hyperlordosis (HY) further decreased foraminal area by 8.9%, as demonstrated in Figure 4, however, these numbers did not yield statistical significance yet (P ≥ 0.05).

Assessment of spinal alignment

**Figure 5:**
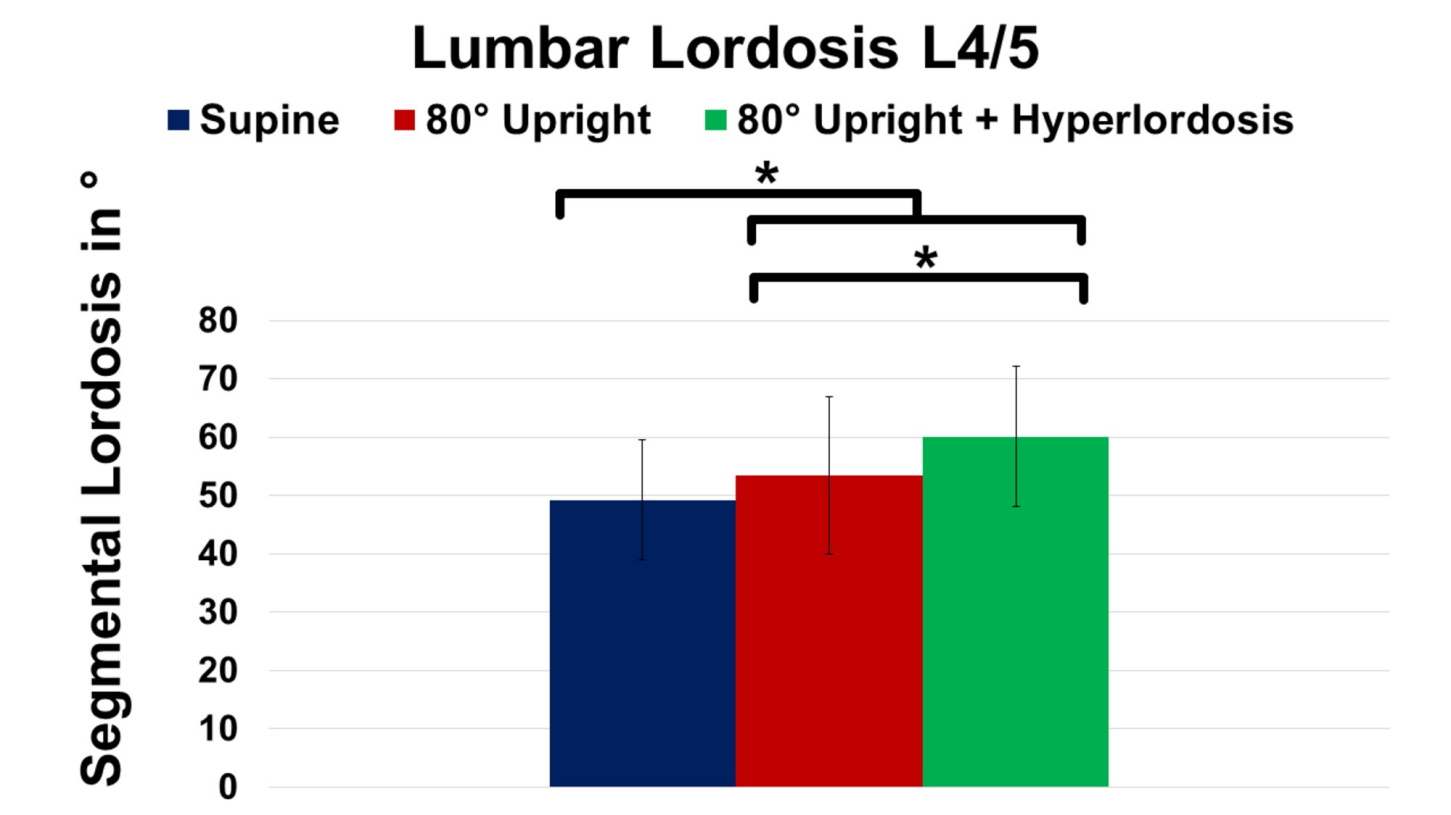
Change of lumbar lordosis at spinal level L4/5 in supine, 80° upright, and 80° upright combined with hyperlordosis position. *P ≤ 0.05

In order to evaluate sagittal spinal alignment, sagittal translation, lumbar lordosis, and segmental listhesis were analyzed at UP, SP, and HY (Table 2). As demonstrated in Figure 5, lumbar lordosis did not reveal a significant increase due to positional modification of MRI imaging between SP and UP (P > 0.05).

**Figure 6:**
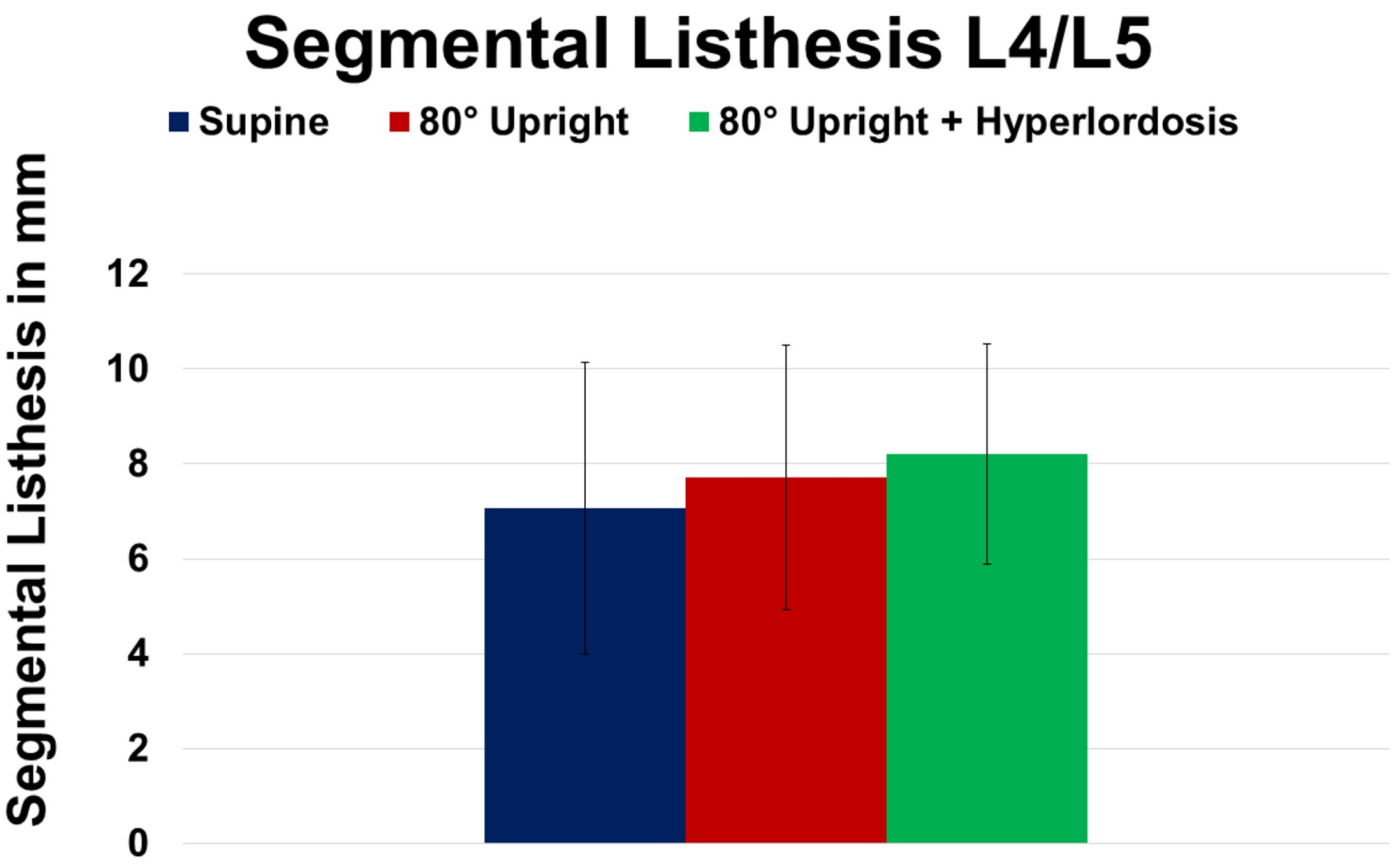
Change of segmental listhesis at spinal level L4/5 in supine, 80° upright, and 80° upright combined with hyperlordosis position.

However, the addition of hyperlordosis caused further improvement of lumbar lordosis yielding significance compared to SP (+22.1%; P = 0.001) as well as to UP (+12.4%; P = 0.002). Additionally, segmental listhesis at L4/5 markedly increased at UP (+9.1%; P ≥ 0.05) and HY (+16%; P ≥ 0.05) compared to SP imaging, respectively (Figure 6).

**Figure 7:**
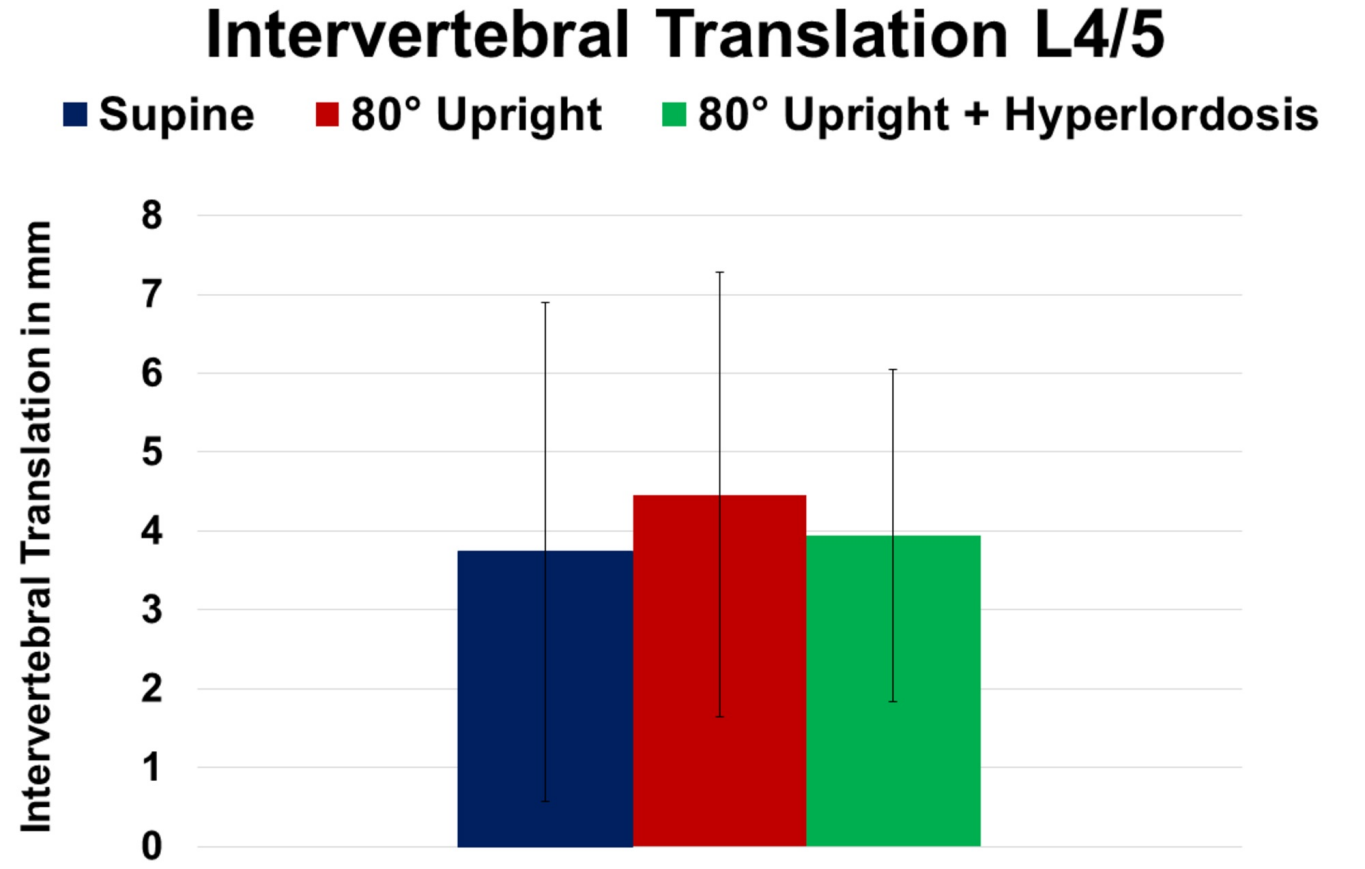
Change of intervertebral translation at spinal level L4/5 in supine, 80° upright, and 80° upright combined with hyperlordosis position.

Addition of HY did not further increase segmental listhesis (Table 2). Finally, sagittal translation did not reveal significant differences when assessed at SP, UP, and during HY imaging (P ≥ 0.05; Figure 7).

## Discussion

The present study sought to assess neural dimensions via dynamic MRI in supine, upright, and upright-hyperlordotic position in order to evaluate the impact of patient positioning on neural narrowing in patients with LSS. Our data suggest that an upright and/or hyperlordotic position in MRI significantly increases foraminal stenosis compared to conventional supine imaging techniques. Neuroforaminal dimensions assessed by supine MRI are potentially overestimated in patients with LSS. Upright MRI imaging may provide the most accurate morphological assessment of neural stenoses in LSS if direct or indirect decompression is considered.

Assessment of neural dimensions via upright MRI

MRI is considered as gold standard for assessing LSS, although there is minimal evidence on the predictive value of MRI findings and their correlation with clinical outcomes following decompression surgery [2]. This might be partially attributed to the lack of a standardized algorithm for evaluating LSS. As current radiological parameters mostly fail to correlate with clinical outcome measures following decompression surgery, a sufficient assessment of neural dimensions preoperatively would be highly beneficial to estimate the required amount of neural decompression. Within this diagnostic gap, upright MRI has gained increasing scientific interest as it allows for physiological load-bearing visualization of spinal elements. Our data and other groups confirm upright MRI to be a non-invasive alternative for the assessment of neural narrowing especially if conventional imaging modalities failed to demonstrate central canal-, foraminal-, or lateral recess stenosis in symptomatic patients. Furthermore, upright MRI permits visualization and extensive assessment of occult disc pathologies in patients with acute or chronic low back pain in its most physiologic condition. Additionally, upright MRI imaging is an alternative for patients who do not tolerate conventional supine MRI chambers due to claustrophobia or other contraindications. Previous studies demonstrated that there is a significant reduction in central canal and lateral recess dimensions in asymptomatic as well as symptomatic subjects assessed by standing compared to supine (and flexion compared to extension) MRI [10, 12-14]. Our data confirm these findings. Other authors observed spinal extension to reduce central canal, lateral recess, and foraminal area whereas spinal flexion increases above mentioned radiographic parameters. In the present study, we found equivalent phenomena (Figures 2-4; Table 2). Schmid et al. observed a 5.2% reduction of central canal area comparing supine and upright MRI imaging in asymptomatic volunteers [15]. When comparing neural dimensions during upright flexion to upright extension MRI, the authors even reported a reduction of central canal area of up to 16.4%. Our data are in accordance with previous findings yielding a reduction of central canal area by 8.3% and 7% due to UP and/or HY imaging modalities (Table 2).

Whereas bulging discs seem to play a minimal role in contributing to LSS in asymptomatic patients, laxity of ligamentum flavum, hypertrophy, and disc degeneration are suggested to significantly impair central canal dimensions in symptomatic patients [14-16]. In the present study, patients neither had primary spondylolysis (pars interarticularis defect) nor disc herniations at the index level to be investigated, revealing that the observed changes on neural dimensions most likely occurred due to anatomical alterations following changes in patient positioning. In the same context, progressive disc degeneration was found to cause increased disc height loss as well as segmental instability in upright compared to supine MRI [12, 17]. Zou et al. analyzed patients having low back pain via kinetic MRI in a neutral weight-bearing position (either standing or sitting) and also in extension and flexion, and found that there was a significant increase in the degree of lumbar disc herniation in flexion and extension when compared with neutral views alone. In fact, extension scans led to significantly higher detection rates compared to flexion imaging (16% vs 12%) and moreover, incidence of missed disc herniations yielded up to 19% when comparing extension MRI to conventional upright MRI and 16% when compared to flexion upright MRI [18]. In summary, clinically relevant spinal canal and neuroforaminal stenosis can be uncovered by imaging in the erect position. In cases where conventional MRI shows no evidence of lumbar central canal or nerve root compression in the setting of convincing clinical symptoms that warrant surgical intervention, reimaging in the upright position, with the addition of flexion and extension, may help to overcome inconclusive clinical/radiographic findings [19]. As neural dimensions undergo position-dependent alterations erect imaging information is highly relevant clinically because foraminal and/or central canal stenosis may be underdiagnosed with regular MRI.

Upright MRI in spondylolisthesis

Patients having spondylolisthesis frequently describe worsening pain following prolonged standing or load bearing. Nevertheless, most commonly, spinal diseases are evaluated with patients in the supine position. Our data and others have demonstrated that lumbar lordosis is reduced in supine position (Table 2). Consequently, the patient’s level of back pain also tends to lessen. Certainly, conventional standing radiographs are a cost-efficient alternative when assessing the level of spondylolisthesis. However, conventional radiographs do not provide further information on potential neural (central-, foraminal-, or lateral recess stenosis) narrowing due to spondylolisthesis. Therefore, the present study sought to assess kinematic dependent changes on neural dimensions via upright MRI. Imaging of the spinal column in an upright position enables for a reliable functional assessment under axial load [12]. Accordingly, our present data reveal that an increasing axial load causes a progressive sagittal translation of the lumbar vertebrae – though our values did not reach significance levels, most likely due to the small number of patients (Figure 6). Therefore, it remains conclusive that the pain experienced by patients with hyperlordotic spondylolisthesis can be aggravated by an increasing load on the facet joints. In this context, Ben-Galim and Reitman have recently introduced the “distended facet sign” (hypertrophy and edema surrounding the facet joints), as an additional hint for occult positional instability in scenarios when MRI may not demonstrate significant stenosis in patients with neurogenic claudication [20].

Upright MRI and lumbar lordosis

Besides affecting neural dimensions and spondylolisthesis, spinal alignment is also influenced by patient positioning during imaging. To date, assessment of sagittal balance and/or spinal alignment is mainly performed via conventional standing X-rays. Though, conventional radiographs fail to provide morphologic information on neural elements. Recently, Brink et al. evaluated spinal morphology and alignment in upright, prone, and supine position in patients with adolescent idiopathic scoliosis [21]. Although the authors observed a relevant underestimation of spinal deformity in supine and prone position compared to upright imaging, a significant correlation of spinal alignment parameters was found among different body positions assessed by different imaging modalities. Additionally, recent research suggests that weight-bearing due to axial rotation, extension, and/or lateral deflection impairs neural processes compared to non-weight-bearing situations [17, 22, 23]. Our study demonstrated that erect posture of patients combined with hyperlordotic positioning (HY) caused a significant increase in lumbar lordosis (+22.1%; P: 0.001) compared to supine position. In LSS patients, HY positioning most commonly translates to increased pain sensation. As long as global spinal balance is maintained, lumbar lordosis (~40-60°) enables impacts affecting the discs to be absorbed and deflected. Furthermore, LSS raises the pressure on the dorsal intervertebral disc and potentially results in ventral displacement of the nucleus pulposus [24, 25]. In an upright position, shear forces are primarily absorbed by the facet joints, whereas the facet joints usually absorb 16% of the craniocaudal force affecting the spinal column [26].

Future perspective: three-dimensional (3D) assessment of neural dimensions combined with upright MRI

Assessment of neural dimensions via MRI is crucial in patients with LSS to evaluate the potential and limitations of decompression surgery. Current studies assessing neural dimensions regularly use two-dimensional (2D) measures such as foraminal diameters to quantify neural compression and decompression before and after surgery. However, 2D measurements may hide the true effects of “ligamentotaxis” and decompression of neural elements inside of the central canal and foramen. 3D volumetric analysis would provide a more accurate representation, and has the potential to provide more accurate predictive criteria for patients undergoing indirect and direct decompression surgery which may better elucidate correlations between radiographic findings and clinical outcomes in LSS. Recently, our group has proposed 3D measurements of intraoperative radiographic parameters in patients undergoing extreme lateral interbody fusion (XLIF) [27]. In this context, expected increases in 2D parameters likely overestimated the actual anatomical changes occurring in XLIF. Due to the ability of a 3D representation to more completely reveal the anatomical changes that occur following cage insertion we believe that volumetric analysis is a better surrogate for assessing neural dimensions pre- and postoperatively. Therefore, we strongly believe that unless we analyze neural narrowing by 3D assessment combined with upright MRI imaging, we still fail to precisely evaluate whether neural decompression is indicated or not. By combination of volumetric neural assessment with upright MRI, the need for subsequent surgeries due to persistent or recurrent symptoms or adjacent segment disease might be reduced. Future studies will focus on the correlation of volumetric measurements and LSS specific symptoms in order to further elucidate the minimal increase in central canal/neuroforaminal volume required for minimal clinical improvement.

Limitations and strengths

Our study is associated with several limitations. Firstly, a lack of a control group, intermediate postoperative and/or follow-up examinations (to assess clinical and functional outcome following surgery) must be mentioned. Also, with a total of 10 patients, our study population is relatively small. However, the present study size is in line with previously reported investigations [11, 28]. Further investigations on the kinematic dependencies of neural narrowing will be carried out in a larger population. Additionally, our study population was biased due to inclusion criteria. Moreover, erect MRI imaging was conducted in 80° but not in 90°. The quasi full upright position was chosen as the HY position was not possible in the full 90° upright posture.

Certainly, we have to admit that availability of upright MRI imaging is currently limited to a few high-volume academic spine centers. Finally, spatial resolution with the MRI utilized in the present study was significantly lower compared to state of the art high-field MRIs. Thus, several studies demonstrated that by adjustment of slice orientation and sequence design high accuracy during functional MRI of the musculoskeletal system can be achieved [29]. To our knowledge, there are no previous human studies on positional alterations of LSS and their impact on neural dimensions and sagittal alignment in LSS at L4/5 utilizing upright MRI imaging.

## Conclusions

Neural dimensions undergo position-dependent alterations. Neuroforaminal dimensions assessed by conventional supine MRI are at risk to be overestimated in patients with LSS. Especially in patients having occult disease not visualized on conventional imaging modalities, upright MRI allows for the most realistic and precise assessment of neural elements in LSS when decompression is considered. Evaluation of neural dimensions in erect imaging is highly significant as LSS may be undiagnosed with regular MRI and surgical intervention without adequate decompression may lead to poor outcomes, potentially requiring revision surgery.
